# Deep Margin Elevation and Lithium Disilicate Overlay Restoration of an Endodontically Treated Molar: A Case Report

**DOI:** 10.7759/cureus.113384

**Published:** 2026-07-26

**Authors:** Amira Yousfi, Sihem Hajjeji, Narjes Hassen

**Affiliations:** 1 Prosthodontics, Faculty of Dental Medicine of Monastir, Monastir, TUN

**Keywords:** 10-mdp, adhesive dentistry, deep margin elevation, endodontically treated teeth, lithium disilicate, overlay

## Abstract

Restoring endodontically treated teeth with deep subgingival margins is challenging due to difficulties in isolation, moisture control, and adhesive cementation. Conventional approaches such as surgical crown lengthening are invasive and may compromise periodontal support. Deep margin elevation (DME) offers a conservative alternative by relocating the cervical margin coronally using composite resin. This case report describes a 38-year-old patient with a repeatedly debonded partial indirect restoration on tooth 47, which had previously undergone endodontic treatment. After removal of the existing restoration and caries removal, a deep occlusoproximal defect with a subgingival cervical margin was identified. DME was performed using a bulk-fill composite resin to reposition the margin supragingivally. The definitive restoration was a lithium disilicate overlay. The procedure appears to offer a conservative approach, preserving dental tissues and periodontal health, and suggests the potential clinical applicability of this technique.

## Introduction

Restoring endodontically treated posterior teeth with deep subgingival margins is one of those clinical situations that can quickly become challenging. The margins are difficult to isolate; they are constantly contaminated by saliva, blood, and gingival crevicular fluid, and recording them with either conventional or digital impressions is rarely straightforward [1]. When it comes to adhesive cementation of indirect restorations, the situation becomes even more complicated; controlling excess cement in subgingival areas is notoriously difficult, and the risk of leaving residual material that could compromise periodontal health is ever present [2]. On top of these technical challenges, deep proximal caries frequently extend beyond the cementoenamel junction, where enamel gradually disappears and the cavity margin consists of dentin and cementum, substrates that are considerably less favorable for bonding [3].

Traditionally, clinicians have turned to surgical crown lengthening, orthodontic forced extrusion, or surgical extrusion to manage these cases. Crown lengthening, while predictable, is an invasive procedure. It adds cost, extends treatment time, and can compromise the crown-to-root ratio, a particular concern for endodontically treated teeth that are already structurally compromised [4]. Orthodontic extrusion is slow, expensive, and depends heavily on patient compliance [5]. Surgical extrusion carries its own risks, including progressive root resorption, and often requires root canal treatment in teeth with mature apices [6]. These interventions are associated with significant clinical limitations. 

This is where deep margin elevation (DME), first described by Dietschi and Spreafico in 1998, offers a compelling alternative [7]. Rather than surgically exposing the margin by displacing the periodontium, DME does the opposite, it brings the margin coronally by placing a composite resin base [2]. The technique is based on a simple principle: relocate the restorative margin rather than the periodontal tissues. By doing so, it preserves the supracrestal tissue attachment while making it possible to achieve the dry, isolated field that adhesive procedures demand [1]. It simplifies impression taking, facilitates cementation, and makes excess cement removal far more predictable [8]. Available evidence suggests favorable long-term outcomes for indirect restorations placed following DME [9]. This case report describes the application of DME in an endodontically treated mandibular second molar, where a repeatedly debonded indirect restoration was replaced by a lithium disilicate overlay.

## Case presentation

A 38-year-old female patient presented for rehabilitation of the mandibular right second molar. The tooth had previously undergone endodontic treatment and had a history of repeated debonding of a bonded indirect partial restoration. The patient's medical history was non-contributory. She was a non-smoker, reported no parafunctional habits, and maintained good oral hygiene with regular dental visits. No relevant medications or systemic conditions affecting healing were reported, and no known allergies were noted.

**Figure 1:**
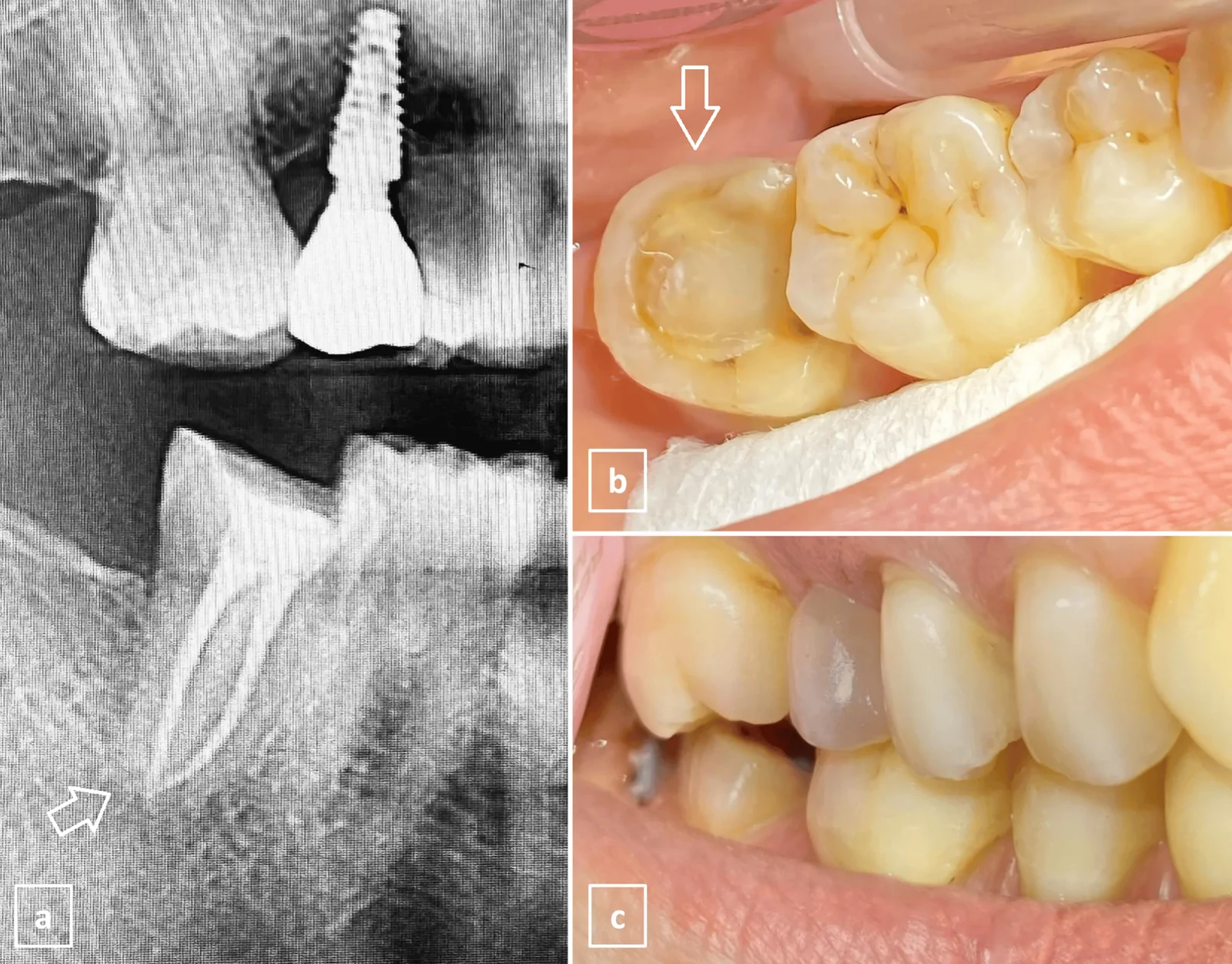
Initial clinical and radiographic examination (a) Preoperative radiograph showing the endodontically treated mandibular right second molar (tooth 47) with adequate root canal filling and absence of periapical pathology. (b) Initial occlusal view of tooth 47 showing the existing composite restoration with recurrent caries at the restoration margin. (c) Initial occlusal relationship between the extruded antagonist tooth and tooth 47.

Clinical examination revealed extrusion of tooth 47 and its maxillary antagonist, resulting in occlusal interferences. Selective occlusal enameloplasty was therefore performed on the opposing tooth to re-establish a harmonious occlusal plane before definitive rehabilitation. Tooth 47 exhibited an extensive composite build-up, which had been placed beneath the debonded indirect restoration, with recurrent caries at the restoration margin (Figure 1b).

The inadequate marginal adaptation of the existing restoration was considered a possible contributing factor to its repeated failure. The preoperative radiograph showed no evident periapical radiolucency. The tooth was clinically asymptomatic, and the existing root canal filling appeared satisfactory on the available radiograph (Figure 1a).

Following rubber dam isolation, the existing restoration and residual composite were carefully removed. Caries excavation was performed using low-speed carbide burs mounted on a contra-angle handpiece until sound dental tissues were obtained. Following complete caries removal, the cervical margin of the mesial proximal box extended below the cemento-enamel junction and remained subgingival, precluding predictable adhesive isolation and restorative procedures.

**Figure 2:**
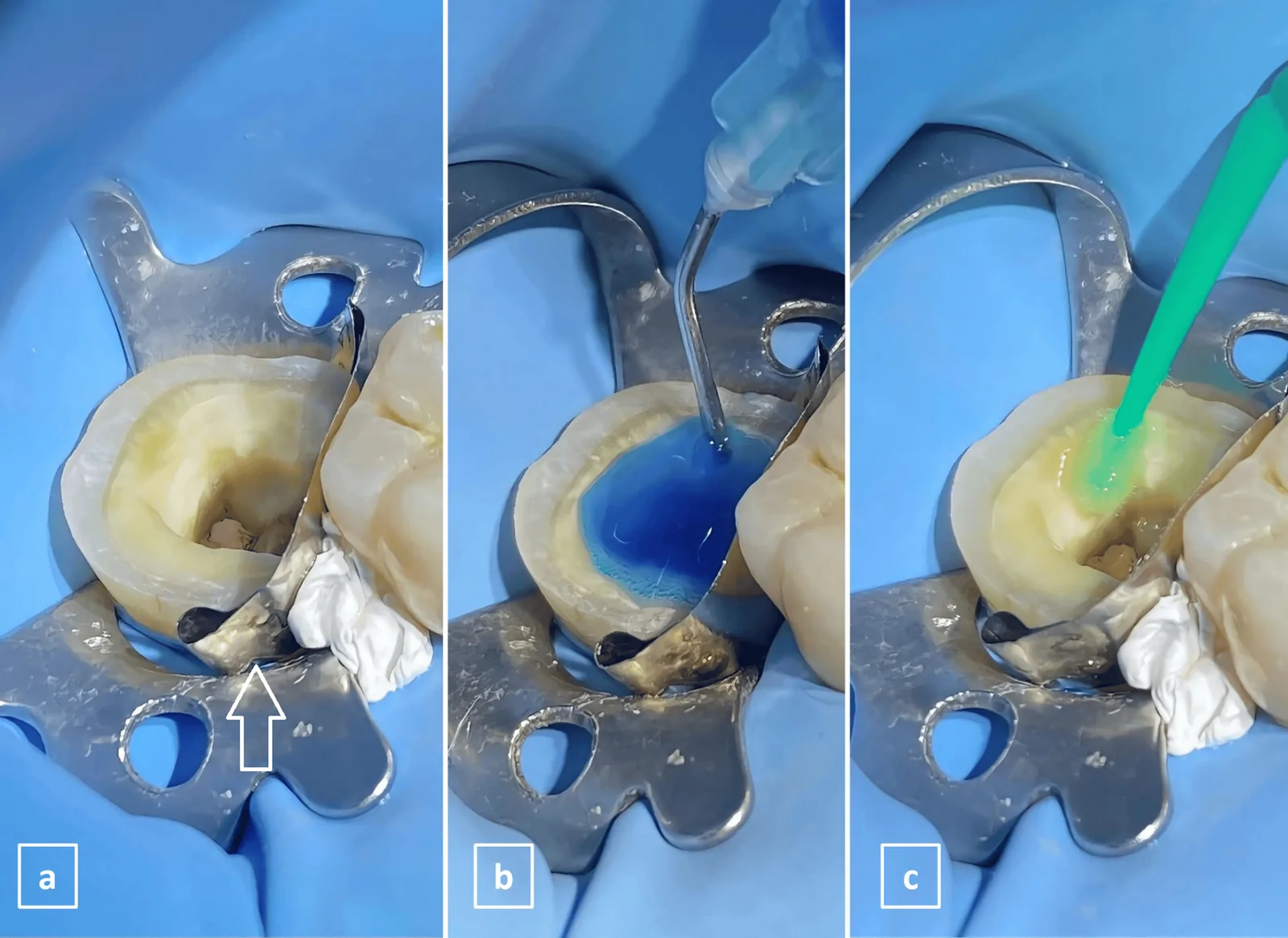
Adhesive procedures performed before deep margin elevation (a) Placement and adaptation of the sectional matrix system at the deep cervical margin. (b) Selective dentin etching with phosphoric acid. (c) Application of the universal adhesive system (All-Bond Universal, BISCO).

A sectional metal matrix band was carefully adapted to the proximal box. A strip of polytetrafluoroethylene (PTFE) tape was used to improve cervical adaptation of the matrix band and obtain intimate adaptation of the deep cervical margin while preventing interposition of the rubber dam between the matrix band and the cavity margin (Figure 2a).

Dentin etching was performed with 37.5% phosphoric acid for five seconds. This shortened etching time was chosen to minimize the risk of over-etching the dentin substrate in the subgingival area, where the margin consisted predominantly of dentin and cementum (Figure 2b). The cavity was then thoroughly rinsed and gently air dried. A universal adhesive containing 10-methacryloyloxydecyl dihydrogen phosphate (10-MDP; All-Bond Universal, BISCO, Schaumburg, IL, USA) was actively rubbed onto the tooth surface for 20 seconds, gently air-thinned, and light-cured for 20 seconds according to the manufacturer's instructions (Figure 2c).

**Figure 3:**
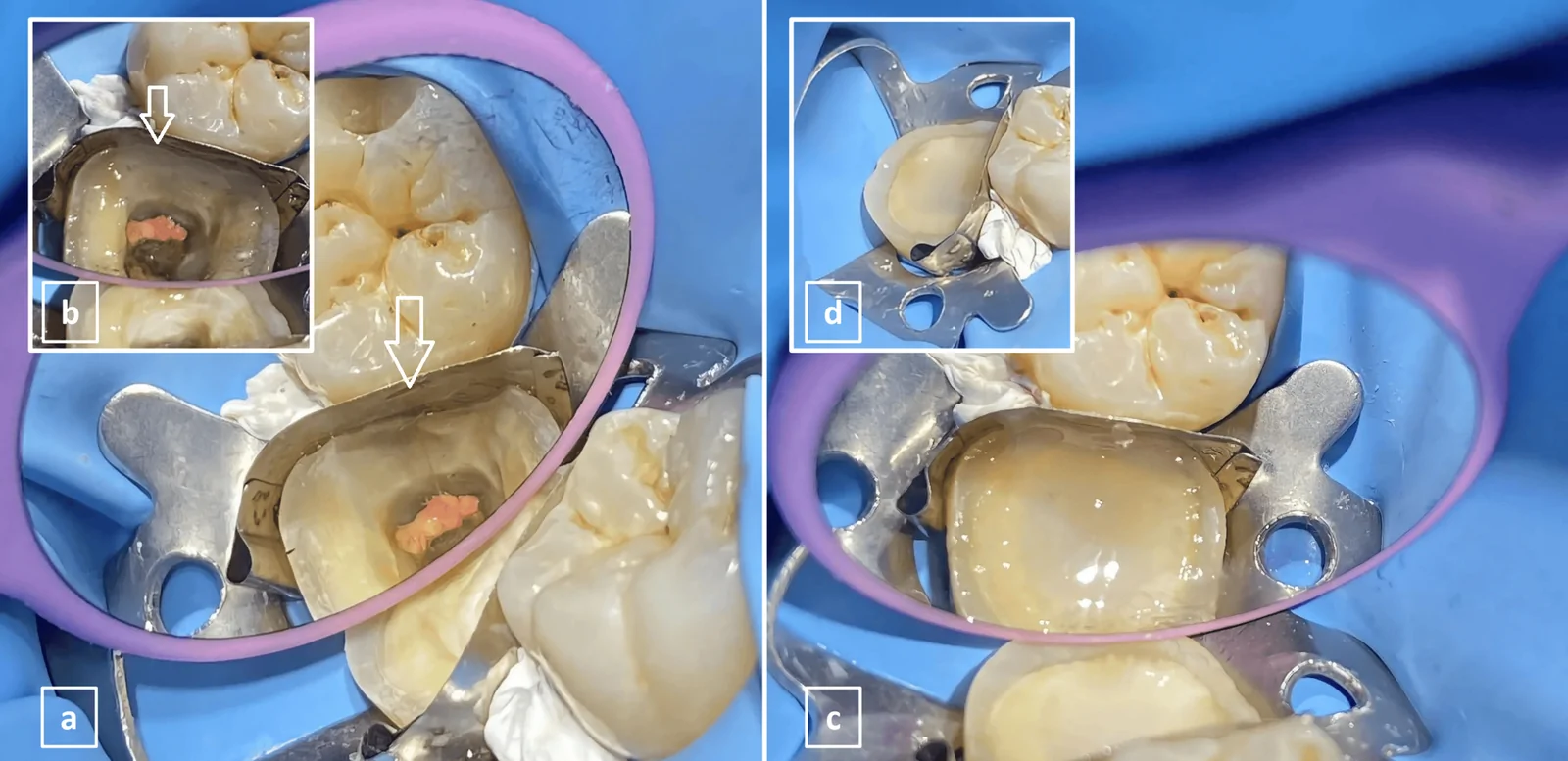
Deep margin elevation procedure and composite build-up (a) Occlusal view showing the adapted sectional matrix system and cervical adaptation at the deep margin. (b) Placement of the first increment of bulk-fill resin composite at the cervical margin to relocate the subgingival margin. (c) Occlusal view after restoration of the remaining cavity with bulk-fill resin composite. (d) Buccal view showing the completed composite build-up after deep margin elevation.

DME was performed using a bulk-fill resin composite to relocate the subgingival cervical margin to a supragingival position. A 2 mm cervical increment was first placed at the deep margin (Figure 3b).

After polymerization, the remaining cavity was restored with the same bulk-fill resin composite according to the manufacturer's recommended curing protocol. The completed composite build-up was evaluated from both occlusal and buccal views, providing a stable bonded foundation for the definitive indirect restoration (Figures 3c, 3d).

**Figure 4:**
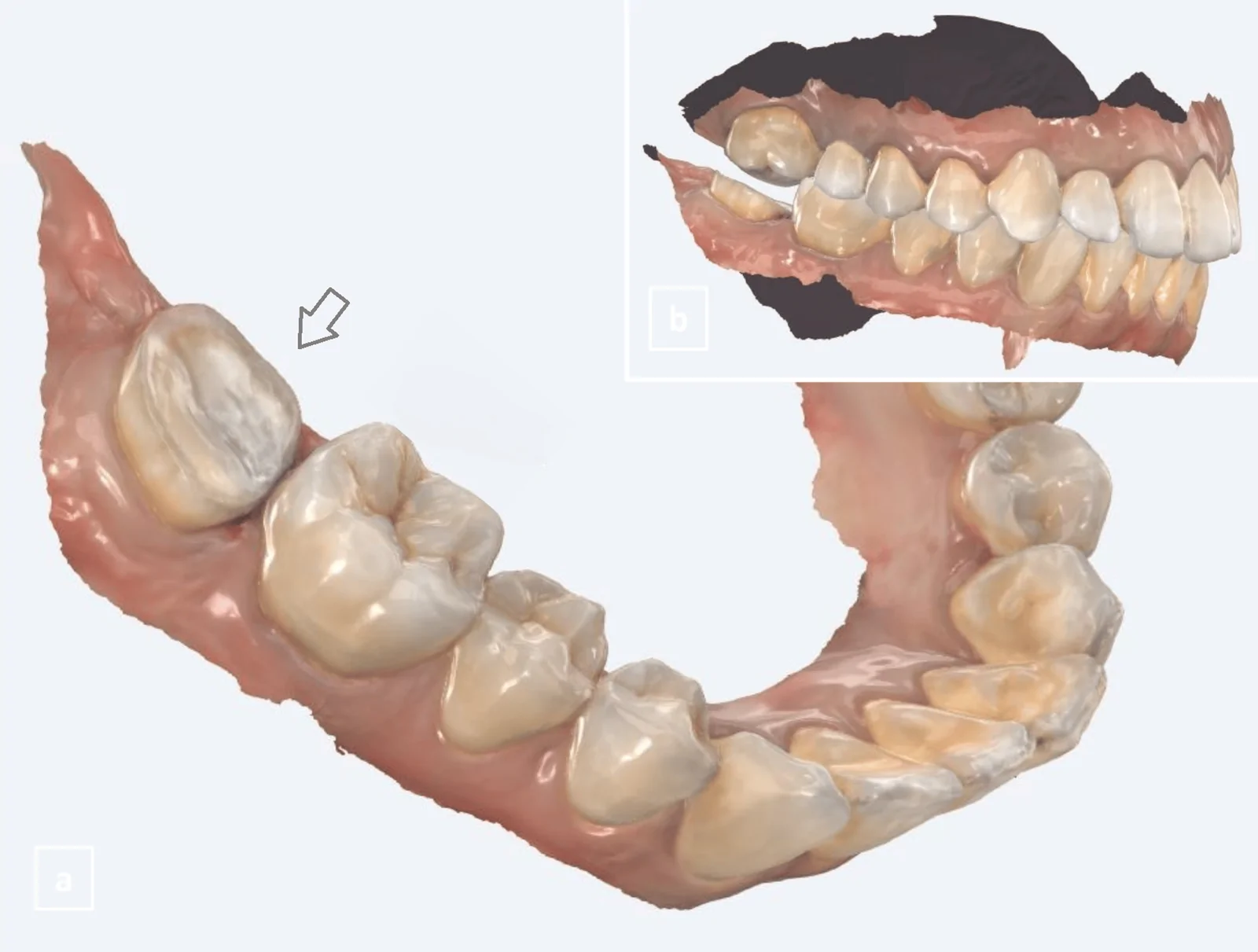
Digital acquisition of the preparation and occlusal relationship (a) Intraoral digital scan of the prepared tooth. (b) Digital recording of the maxillomandibular occlusal relationship.

The tooth was subsequently prepared to receive a lithium disilicate overlay restoration. A digital impression of the preparation and the opposing arch was obtained using an intraoral scanner Medit i600, and the maxillomandibular relationship was digitally recorded (Figures 4a, 4b).

**Figure 5:**
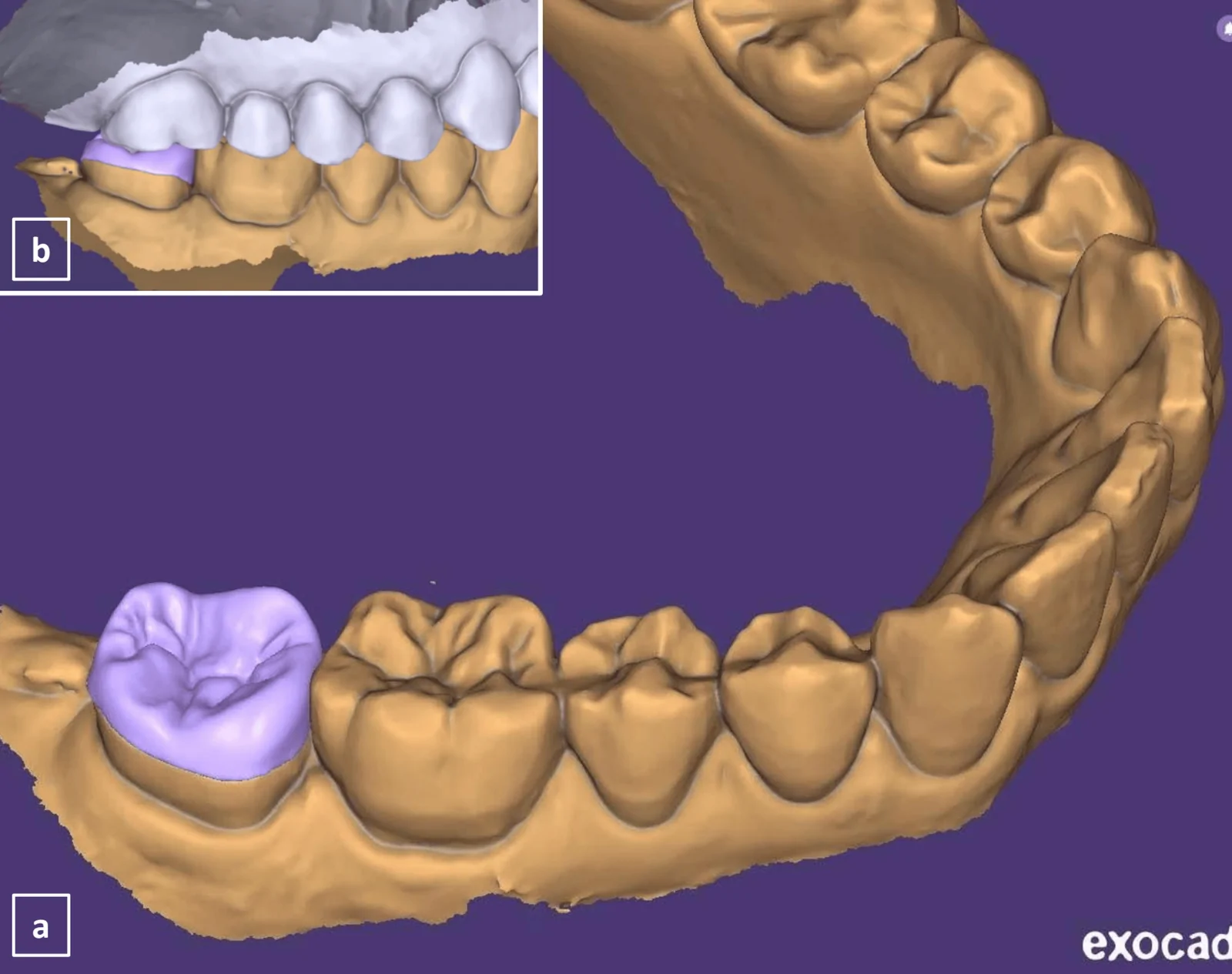
Digital workflow for the design and virtual occlusal assessment of the lithium disilicate overlay restoration (a) Digital design of the lithium disilicate overlay using exocad software. (b) Virtual evaluation of the overlay restoration in occlusion.

The lithium disilicate overlay was virtually designed using exocad software, and the occlusal relationship was evaluated digitally before fabrication (Figures 5a, 5b).

**Figure 6:**
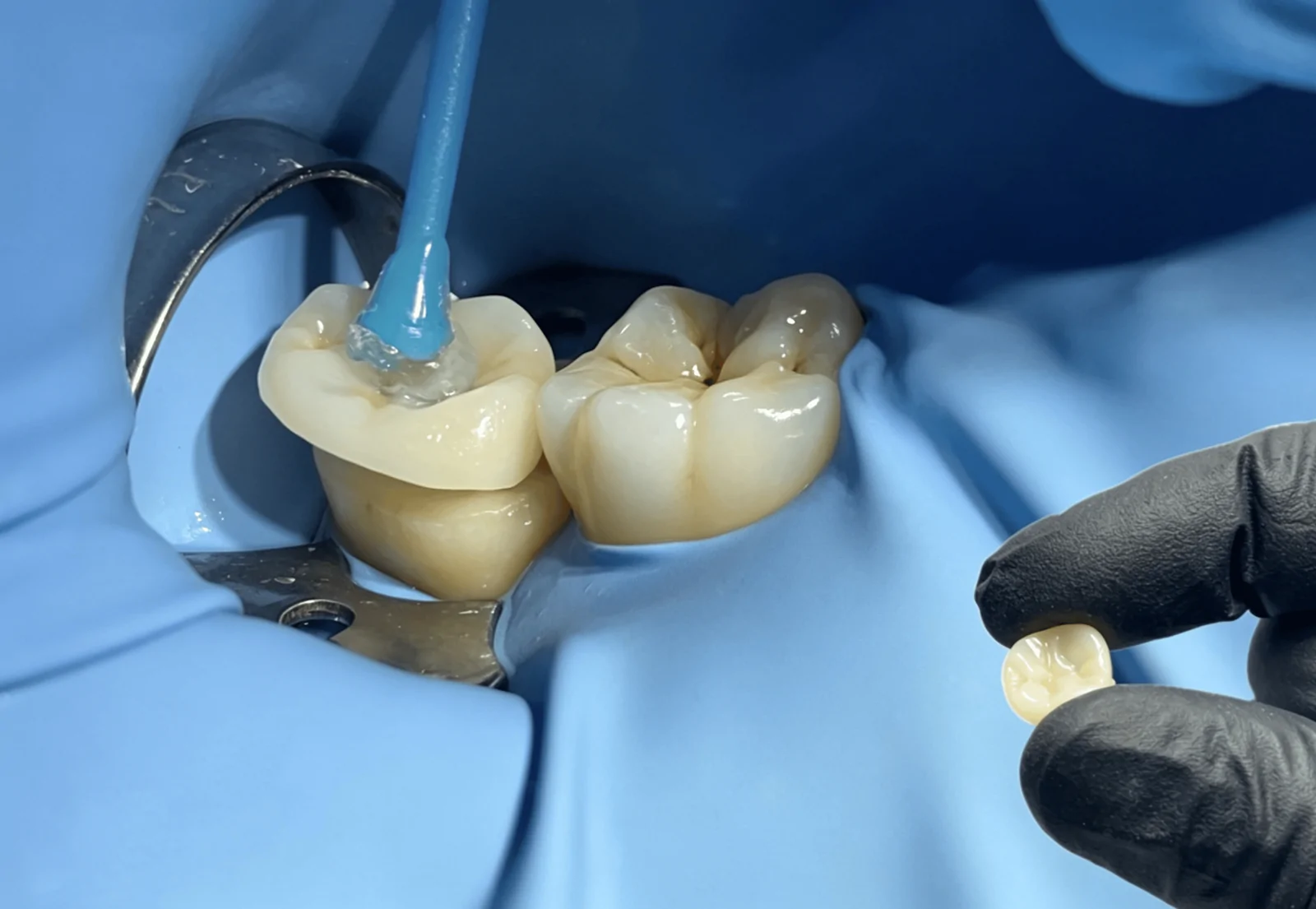
Clinical try-in of the lithium disilicate overlay restoration under rubber dam isolation

The restoration was then tried intraorally to assess marginal adaptation, proximal contacts, and occlusion. Following the try-in procedure, the rubber dam was applied, and the adaptation of the overlay restoration was re-evaluated under isolated conditions before proceeding with the adhesive cementation protocol (Figure 6).

**Figure 7:**
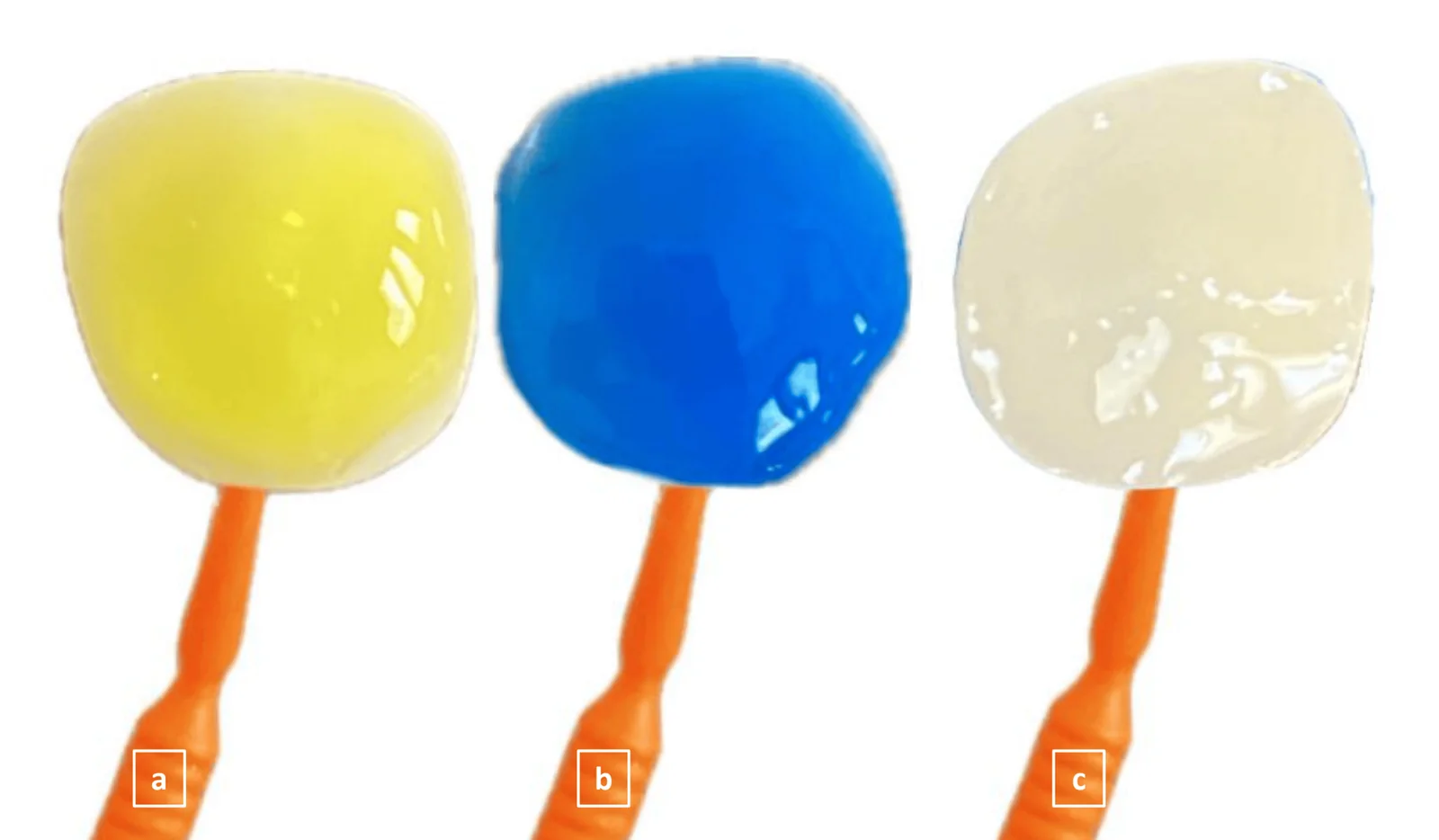
Surface treatment of the lithium disilicate overlay before adhesive cementation (a) Etching of the intaglio surface with hydrofluoric acid. (b) Application of phosphoric acid to remove residual fluorosilicate precipitates after hydrofluoric acid etching. (c) Application of the silane coupling agent.

The intaglio surface of the lithium disilicate overlay was etched with 9% hydrofluoric acid for 20 seconds, thoroughly rinsed, and air dried. Thirty-seven percent phosphoric acid was then applied to remove the residual fluorosilicate precipitates generated during hydrofluoric acid etching, followed by rinsing and air drying. A pre-hydrolyzed single-component silane coupling agent (Porcelain Pre-Hydrolyzed Silane, BISCO, Schaumburg, IL, USA) was subsequently applied according to the manufacturer's instructions (Figures 7a-7c).

**Figure 8:**
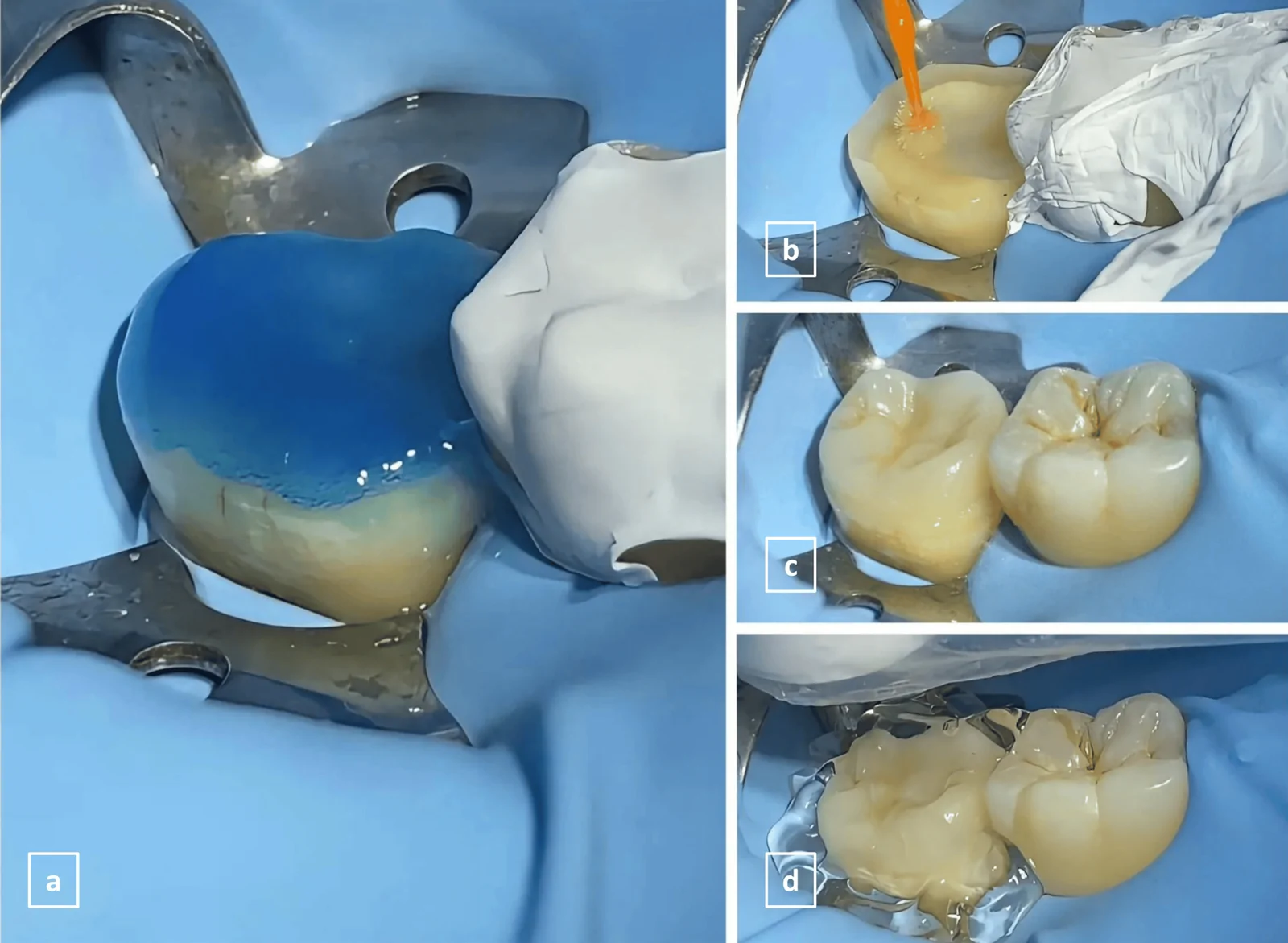
Adhesive cementation protocol of the lithium disilicate overlay restoration (a) Etching of the tooth surface with 37% phosphoric acid. (b) Application of the universal adhesive system (All-Bond Universal, BISCO). (c) Placement of the lithium disilicate overlay filled with dual-cure resin cement (Duo-Link Universal, BISCO) onto tooth 47. (d) Final light polymerization performed under a glycerin gel layer to eliminate the oxygen-inhibited layer.

The prepared tooth surface was etched with 37% phosphoric acid, rinsed, and gently air dried (Figure 8a). All-Bond Universal adhesive (BISCO, Schaumburg, IL, USA) was actively applied, gently air-thinned, and light-cured for 20 seconds (Figure 8b). The restoration was adhesively luted using a dual-cure resin cement (Duo-Link Universal Resin Luting Cement, BISCO, Schaumburg, IL, USA) under constant seating pressure (Figure 8c). Excess cement was removed during the gel phase, and a layer of glycerin gel was applied around the restoration margins before final light polymerization to limit oxygen contact and facilitate polymerization of the superficial resin layer (Figure 8d).

**Figure 9:**
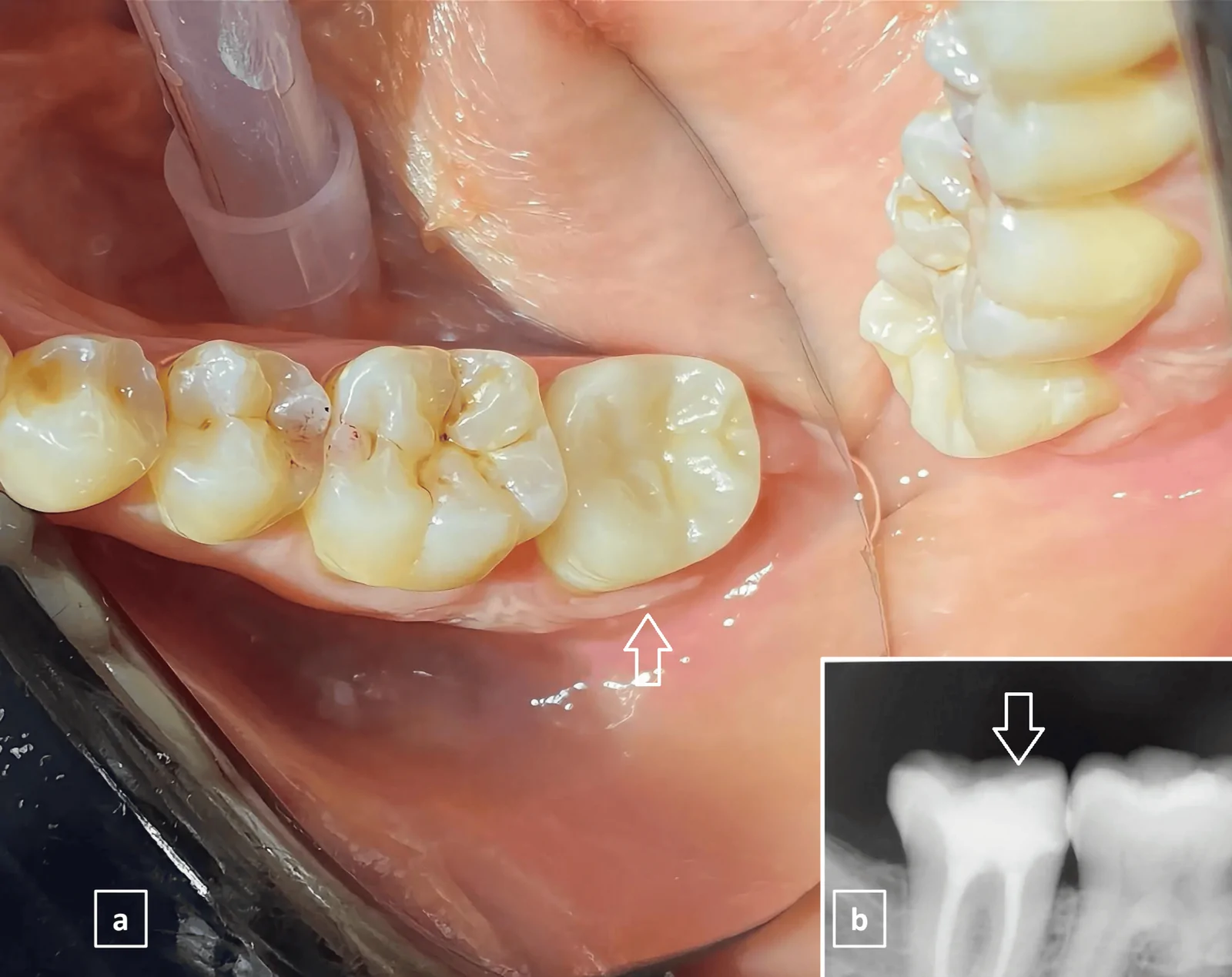
Final restoration and radiographic evaluation (a) Occlusal view of the adhesively bonded lithium disilicate overlay restoration on tooth 47. (b) Bitewing radiograph demonstrating the marginal adaptation of the deep margin elevation area and the overlay restoration.

Occlusion was carefully verified and adjusted where necessary, followed by final polishing of the ceramic restoration using a dedicated ceramic polishing system. The completed adhesively bonded lithium disilicate overlay restoration was then clinically evaluated from the occlusal view (Figure 9a).

A postoperative bitewing radiograph was obtained to assess the adaptation of the DME area and the marginal integrity of the overlay restoration (Figure 9b).

The patient received postoperative instructions regarding oral hygiene and maintenance and was scheduled for a routine clinical follow-up several months later.

## Discussion

The mesial margin in this case extended below the cementoenamel junction, which created the kind of restorative predicament that often leads clinicians to consider surgical crown lengthening. However, given that the tooth was already endodontically treated and had a history of debonding, osseous resection to expose the margin was considered inadvisable.

DME offered a way to address the access and isolation problems without compromising periodontal support. By relocating the margin coronally with composite resin, we were able to create the dry, isolated field necessary for reliable adhesive bonding while preserving the existing supracrestal tissue attachment [1,2].

This aligns with the fundamental principle of the technique: moving the margin rather than the periodontium. The decision to proceed with DME was not taken lightly. Three conditions had to be satisfied before proceeding: complete isolation of the operating field, a matrix that could achieve a fluid-tight seal, and assurance that the connective tissue component of the attachment would not be violated [1]. In this case, all three were achievable, which gave us confidence that the procedure would be predictable. It is worth acknowledging, however, that DME is highly technique-sensitive. The outcome depends not only on the operator's skill but also on the choice of materials and strict adherence to adhesive protocols [1,8]. When these elements are not optimally managed, the risk of failure increases substantially.

The periodontal response to DME merits consideration. The supracrestal tissue attachment, composed of the junctional epithelium and connective tissue, has been described as a zone that should not be violated [10]. What makes DME biologically acceptable is that the restoration does not invade the connective tissue attachment, it simply occupies space within the junctional epithelium, which is capable of reforming along smooth restorative surfaces. Histological studies have confirmed that epithelial cells can establish hemidesmosomal attachments to well-polished composite resins, which helps explain why DME restorations are generally well-tolerated [11]. Clinical studies have shown that subgingival composite restorations can be compatible with gingival health, producing inflammatory responses similar to those seen on untreated root surfaces [12]. However, the distance from the restoration margin to the alveolar bone crest appears to be a critical determinant of periodontal health. Ferrari et al. evaluated the periodontal response to cervical margin relocation (CMR) and reported a significantly higher incidence of bleeding on probing (BoP) when margins were placed at or closer than 2 mm from the bone crest [13]. In the present case, the margin was elevated to a supragingival position, ensuring that the required distance from the bone crest was maintained. This likely contributed to the absence of periodontal complications during the follow-up period.

The adhesive strategy deserves discussion because it was central to the success of this case. Subgingival margins present a bonding challenge primarily because the substrate is dentin and cementum rather than enamel. Enamel provides the most reliable adhesion, while dentin bonding is more technique-sensitive and less durable over time [3]. To address this, we used immediate dentin sealing, which involves applying adhesive resin to freshly cut dentin before taking the impression [14]. This approach has been shown to enhance bond strength, reduce microleakage, and minimize postoperative sensitivity by protecting the dentin from contamination during the provisional phase.

The choice of a 10-MDP-containing universal adhesive was deliberate. MDP is a functional monomer that forms stable, water-insoluble calcium salts with hydroxyapatite through a nano-layering mechanism, creating an adhesive interface that resists hydrolytic degradation [15]. A comprehensive meta-analysis by Fehrenbach et al. found that 10-MDP-based adhesives produce superior bond strength to dentin compared to adhesives formulated with other acidic monomers [16].

Composite resin remains the material of choice for DME because it provides reliable adhesion to tooth structure, favorable mechanical properties, and can be finished to a smooth surface that facilitates periodontal maintenance [1,8]. Different categories of resin composites, including conventional, flowable, and bulk-fill materials, have been proposed for DME. Although some in vitro studies have suggested that certain placement techniques, such as incremental layering, may improve marginal quality compared with bulk placement [17], the available evidence remains insufficient to establish the superiority of one restorative material or technique over another [1,8]. In the present case, a bulk-fill resin composite was selected for both cervical margin relocation and restoration of the remaining cavity because it simplified the restorative procedure while providing adequate depth of cure and satisfactory marginal adaptation in a deep proximal cavity [8].

The definitive restoration was a lithium disilicate overlay. This material offers several advantages for endodontically treated molars: adequate flexural strength for posterior applications, etchability with hydrofluoric acid, and the ability to be silanized for reliable adhesive bonding [18]. When adhesively cemented, lithium disilicate overlays reinforce the remaining tooth structure and distribute occlusal forces favorably, reducing the risk of catastrophic fracture [19]. The overlay design also conserves tooth structure compared to a full crown, which is particularly relevant for teeth that have already lost substantial coronal tissue.

Collectively, these observations support the potential of DME as a primary option in appropriately selected cases. When performed to a high standard, the technique may offer a reliable and minimally invasive alternative to surgical crown lengthening, avoiding the disadvantages associated with osseous resection and preserving the supracrestal tissue attachment. Success, however, depends on strict adherence to established protocols, including complete isolation, proper matrix adaptation, and careful composite placement [1].

## Conclusions

DME offers a conservative and effective restorative option for teeth with deep subgingival defects. This approach preserves tooth structure while enabling reliable adhesive restoration, provided that meticulous technique and appropriate material selection are applied. Periodontal health can be maintained when the procedure is executed with strict adherence to clinical protocols, including proper isolation, matrix adaptation, and respect for the supracrestal tissue attachment. Existing clinical trials support the use of DME, though the available evidence remains heterogeneous. Further long-term studies and randomized controlled trials are needed to establish definitive clinical guidelines and strengthen the evidence base for this technique.
